# Copper-free click chemistry for attachment of biomolecules in magnetic tweezers

**DOI:** 10.1186/s13628-015-0023-9

**Published:** 2015-09-25

**Authors:** Jorine M. Eeftens, Jaco van der Torre, Daniel R. Burnham, Cees Dekker

**Affiliations:** Department of Bionanoscience, Delft University of Technology, Kavli Institute of Nanoscience Delft, Delft, The Netherlands

**Keywords:** Magnetic tweezers, Copper-free click chemistry, SPAAC reactions, Surface chemistry, DNA immobilization

## Abstract

**Background:**

Single-molecule techniques have proven to be an excellent approach for quantitatively studying DNA-protein interactions at the single-molecule level. In magnetic tweezers, a force is applied to a biopolymer that is anchored between a glass surface and a magnetic bead. Whereas the relevant force regime for many biological processes is above 20pN, problems arise at these higher forces, since the molecule of interest can detach from the attachment points at the surface or the bead. Whereas many recipes for attachment of biopolymers have been developed, most methods do not suffice, as the molecules break at high force, or the attachment chemistry leads to nonspecific cross reactions with proteins.

**Results:**

Here, we demonstrate a novel attachment method using copper-free click chemistry, where a DBCO-tagged DNA molecule is bound to an azide-functionalized surface. We use this new technique to covalently attach DNA to a flow cell surface. We show that this technique results in covalently linked tethers that are torsionally constrained and withstand very high forces (>100pN) in magnetic tweezers.

**Conclusions:**

This novel anchoring strategy using copper-free click chemistry allows to specifically and covalently link biomolecules, and conduct high-force single-molecule experiments. Excitingly, this advance opens up the possibility for single-molecule experiments on DNA-protein complexes and molecules that are taken directly from cell lysate.

## Background

Single-molecule methods have become increasingly popular to study biomolecules [1]. With techniques such as atomic force spectroscopy, or optical or magnetic tweezers, one is able to study the mechanical properties of single DNA molecules, single proteins, or individual DNA-protein complexes. The effect of applied force on biomolecules is a particularly relevant topic, as mechanical forces play a crucial role in many cellular processes [2–4]. The relevant forces range from a few pN, like the force produced by an RNA polymerase during transcription (14pN) [5], to tens of pN, as in, for instance, viral packaging motors that use forces of 40pN to compact genomes [6]. Even higher forces are needed in the process of chromosome segregation in eukaryotic cells, where microtubules pull on sister chromatids to segregate them to opposite sides of the spindle pole [7–10]. Many studies using magnetic tweezers have been published that probe the behavior of DNA-protein complexes under applied force and torque [11–16]. For studying biomolecules across the full relevant force range, it is necessary to also measure at higher forces (>20pN). In this regime, however, many traditional anchoring methods fail, thus limiting such single-molecule experiments.

**Fig. 1:**
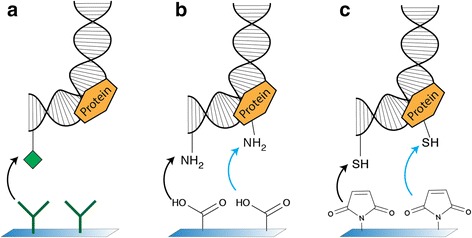
Common DNA tethering techniques. **a** Binding of a digoxygenin-functionalized DNA-protein complex to an anti-digoxygenin-coated surface. This reaction is specific, but unstable when high forces are applied. **b** Binding of an amine-functionalized DNA-protein complex to a carboxyl-coated surface. Both the functionalized DNA (black arrow) and native lysine groups in the protein (blue arrow) bind the surface. **c**. Binding of a thiol-functionalized DNA-protein complex to a maleimide-coated surface. Both the functionalized DNA (black arrow) and native cysteine groups in the protein (blue arrow) bind the surface

For efficient tethering of biomolecules, it is essential to use orthogonal anchoring chemistries on both ends of the molecule, i.e. at the surface and at the bead. To achieve this, a DNA molecule is constructed that has different reactive groups incorporated, on both ends. To complete the anchoring, the bead and surface are functionalized with the corresponding reacting group. A commonly used technique is the binding of biotin to streptavidin. The bond between these functional groups has been shown to resist forces of 150pN [17, 18]. This is a high rupture force compared to a second commonly used method; the binding of a digoxygenin (dig) functionalized nucleotide and a surface coated with antibodies against digoxygenin (anti-dig) (Fig. 1a). This forms a stable non-covalent bond, but a limitation of this binding technique is its low stability under an applied force [19]. Depending on the force-loading rate applied to such a molecule, the dig/anti-dig bond breaks at around 20pN.

Other, much stronger, anchoring methods have been developed [20–24] by functionalizing DNA with amine (Fig. 1b) or thiol groups (Fig. 1c) that are covalently linked to the surface or bead. Although these bonds indeed resist high forces, these techniques have an important limitation in that significant nonspecific binding occurs when studying systems that are more complicated than bare DNA. For example, when studying proteins, native lysines (amine) or cysteines (thiol) in the protein can bind nonspecifically (blue arrows in Fig. 1b, c). For controlled single-molecule measurements, it is however important that the force is being applied at a consistent and known location [25].

A new and exciting challenge is to study DNA-protein complexes that are extracted from cell lysate. For controlled single-molecule experiments, it is essential to anchor these complexes in a stable, strong, and specific way. As the anchoring methods developed so far are unsuitable, studying DNA-protein complexes or complexes from cell lysate remains challenging [26].

Here, we present a novel method for covalent attachment of a DNA tether to a surface, based on copper-free click chemistry. Click reactions are defined as those that are selective, with favorable reaction kinetics, a high yield, and good physiological stability. Early click chemistry reactions required copper as a catalyst [27]. Copper is cytotoxic and thus limits application of click reactions in cells. More recently, copper-free methods became available, for instance the Strain Promoted Azide-Alkyne Click (SPAAC) reaction, of which the reaction between dibenzocyclooctyl (DBCO) and azide is an example [28]. These click reactions are bio-orthogonal, i.e. they can occur within organisms without interfering with native biochemical processes.

**Scheme 1:**
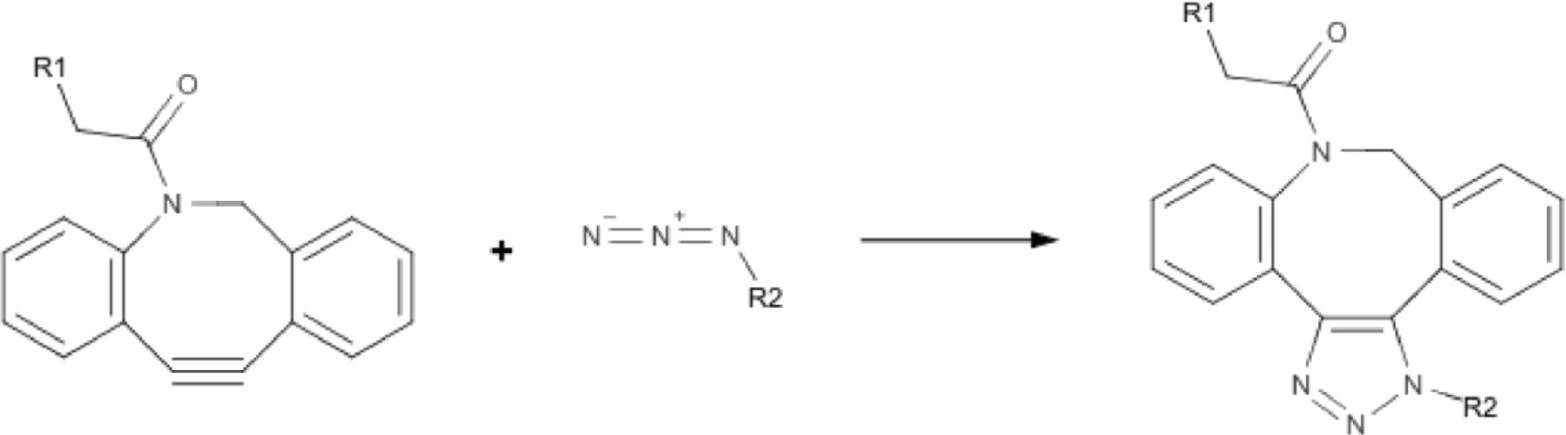
Cycloaddition between dibenzocyclooctyl and azide

As mentioned above, a specific and high-force-compatible anchoring technique is essential for studying DNA-protein complexes in magnetic tweezers. The reactions have to be specific, biocompatible, and able to withstand experimental conditions such as an applied high force. We develop a novel technique for covalent attachment that meets these criteria using copper-free click chemistry, based on the reaction of DBCO with azide (Scheme 1). By functionalizing DNA with DBCO on one end (R1), we can covalently link it to an azide-functionalized surface (R2). As we will show below, this protocol results in a high-yield of DNA tethers, that are torsionally constrained and able to withstand very high forces (>100pN). This method is thus found to be suitable for specifically anchoring DNA-protein complexes and measuring in the relevant force regime.

## Methods

### Magnetic tweezers

**Fig. 2:**
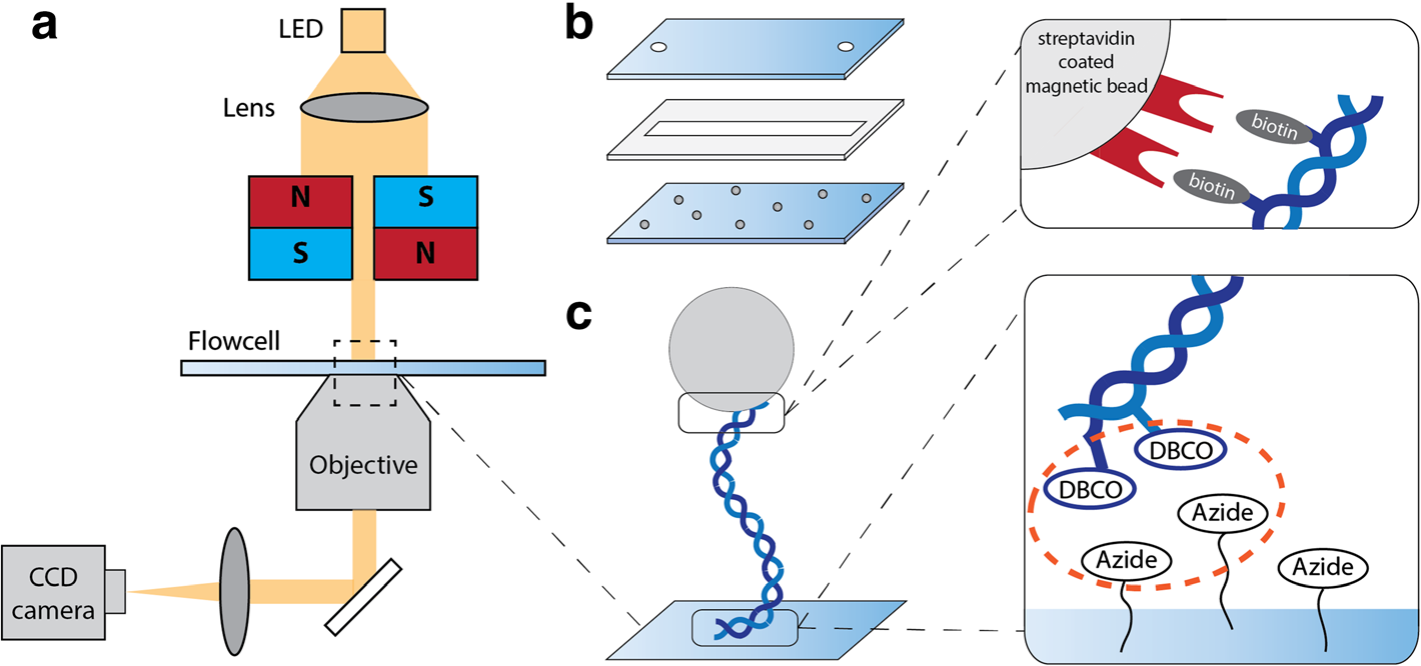
Magnetic tweezers set-up for measuring on a tethered DNA molecule. **a** Schematic of the set-up. A LED illuminates the flow cell through a lens and the magnet holder. Imaging is done with a 50x Nikon objective onto a CCD camera. Magnets manipulate a magnetic bead attached to the DNA. **b** A flow cell is constructed with 24x60mm coverslips. The bottom coverslip is amine-coated and has reference beads bound to it. The top coverslip has sandblasted holes to allow fluid flow. Parafilm is used to seal the coverslips and to create a ˜50 μl flow cell volume. **c** Schematic of a tethered DNA molecule. A DNA molecule is linked to a streptavidin-coated magnetic bead with biotin, and to azide groups on the surface with DBCO at the other end

We used multiplexed magnetic tweezers [29], as illustrated in Fig. 2a. Two 5 mm cube magnets (Supermagnete, N50) are mounted in vertical orientation [30], with a very small (0.3 mm) gap in between them. A red LED provides illumination through the magnet holder onto the flow cell. We use a 50x objective (Nikon) with an achromatic doublet tube lens (200 mm) to provide 50x magnification and image the focal plane onto a CCD camera (Dalsa Falcon 4 M60). Beads are tracked in real time with custom software (Labview, National Instruments) and images are also saved for later analysis [31]. Reference beads are used to correct for drift. The applied force is determined from the Brownian motion of the magnetic bead [32, 33]. For force-extension curves, we perform dynamic force microscopy where the force is increased over time with a constant loading rate of 1 pN/second.

### DNA constructs

A 20678 bp pSupercos1 plasmid was made by removal of the MluI fragment from pSupercos1 (Stratagene) and insertion of two lambda fragments. This Plasmid DNA was isolated with midiprep (Qiagen), restricted with XhoI and NotI.HF (New England Biolabs), and purified (Wizard® SV Gel and PCR Clean-Up System, Promega), resulting in a 20 kb fragment.

DBCO and biotin labeled handles were prepared by PCR on a pbluescriptIISK+ template (Stratagene) with a taq polymerase (GoTaq, Promega) and the addition of Biotin-16-dUTP (Roche), or 5-DBCO-dUTP (Jenabioscience) to the nucleotide mixture respectively. The forward primer was: GACCGAGATAGGGTTGAGTG, and reverse primer: CAGGGTCGGAACAGGAGAGC. The biotin-handle was digested with XhoI resulting in 554 bp and 684 bp fragments. The DBCO-handle was digested with NotI.HF resulting in 624 bp and 614 bp fragments. The handles were purified (Wizard® SV Gel and PCR Clean-Up System, Promega), combined with the restricted plasmid DNA and ligated with T4 DNA ligase (Promega) overnight at 16 °C. The tweezer-construct was then purified again (Wizard® SV Gel and PCR Clean-Up System, Promega).

### Surface functionalization and flow cell assembly

For making amine-coated flow cells, coverslips (Menzel Glaser, 24x60mm, thickness #1) were cleaned in an O_2_ plasma cleaner for 30 s, which ensures activation of the silanol groups. Coverslips were then treated with 2 % APTES in acetone for 10 min, rinsed with MilliQ and air-dried. Before flow cell assembly, polystyrene beads (Polysciences Europe GmbH) were pipetted onto the coverslip and spread with the side of a pipette tip. These non-motile surface-bound beads serve as reference beads for drift correction. The amine-coated coverslips were then aligned with a pre-cut parafilm gasket and another coverslip (Fig. 2b). The assembled flow cell was put on a hot plate at 90 **°**C until the parafilm was sufficiently melted to prevent fluid leakage. The applied heat also firmly binds the polystyrene reference beads to the surface.

### DNA Anchoring

**Fig. 3:**
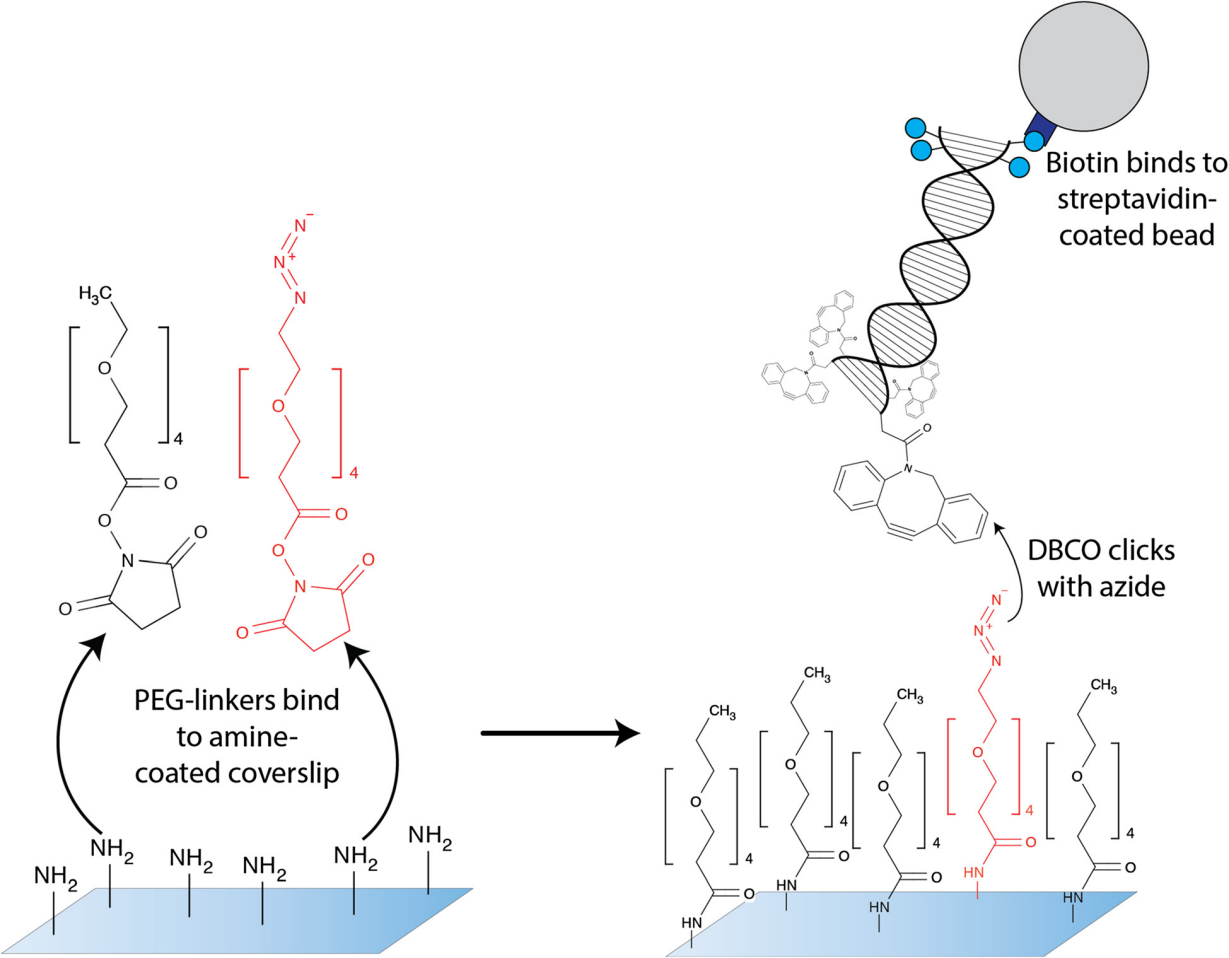
Stepwise linkage of DNA to the surface with copper-free click chemistry. Bifunctionalized PEG-linkers are attached to an amine-coated surface via their NHS group. The NHS ester on the PEG conjugates to the amine on the surface. Non-reactive PEG linkers (terminated with a CH_3_-group) are used to passivate the surface. Finally, a DBCO group on DNA clicks with the azide and thus forms a covalent bond between the DNA and the surface

To anchor the DBCO-functionalized DNA to the amine-coated flow cell, we used bifunctionalized PEG_4_-linkers with an N-hydroxysuccimide (NHS) ester on one end and an azide group on the other (CLK-AZ103, Jenabioscience GmbH, Germany). We mixed azide-functionalized PEG-linkers with CH_3_-terminated PEG-linkers of the same length (MS(PEG)4, Life technologies) in PBS buffer to passivate the surface and prevent aspecific binding. Both PEG-linkers were dissolved in DMSO before further diluting in PBS. To prevent hydrolysis of the NHS ester, the PEG mixture in PBS was prepared shortly before filling the flow cell via capillary action through pipetting the fluid into one flow cell hole of the amine-coated flow cell. The MS-PEG-linker concentration was held constant at 50 mM, while the Azide-PEG concentration was varied (0-50 mM). PEG-linkers incubated in the amine-coated flow cell for 1 h at room temperature, to allow the NHS-ester group to attach to the amine groups in the flow cells (Fig. 3). Next, the flow cell was flushed with washing buffer (20 mM Tris, 5 mM EDTA, pH7.4), to stop the reaction and remove excess PEG. Streptavidin-coated beads (M270 Streptavidin coated, Life Technologies) were incubated with the biotin-functionalized DNA for 20 min. After incubation, the beads were washed 3 times with washing buffer with 0.05 % Tween. An overabundance of DNA-bound beads was then dissolved in 50 μl washing buffer with 0.05 % Tween and flushed into the flow cell. Beads were incubated for 1 h, to allow the DBCO to click with the azide (Fig. 3). Finally, the flow cell was washed with washing buffer until no more unbound beads were visible.

### Control experiment

For control experiments, we used a dig-functionalized DNA construct. The dig handle was constructed in the same matter as the DBCO handle described above, but instead dig-11-dUTP was used (Digoxygenin-11-dUTP, Roche). Coverslips were cleaned in acetone for 30 min in a sonicator for creating the flow cells. After air-drying, they were coated with 1 % nitrocellulose (Invitrogen) in amylacetate (Sigma Aldrich). Application of reference beads and assembly of flow cells proceeded as described above. Next, nitrocellulose-coated flow cells were incubated with 100 mM anti-dig antibodies (Fab-fragment, Roche) for 30 min. After washing as described above, the surface was passivated with 10 mg/ml BSA (Bioke) for 1 h. Preparation of beads proceeded as described above. Beads with digoxygenin-functionalized DNA then incubated in the flow cell for 10 min. Finally, the flow cell was washed with washing buffer until no more unbound beads were visible.

## Results and Discussion

We developed a protocol to covalently attach biomolecules in a magnetic tweezers flow cell using copper-free click chemistry. As described in Methods, we coat the glass surface with azide-functionalized PEG-linkers, and attach DBCO-tagged DNA through the azide-group, thereby covalently linking the DNA molecule at one end to the surface.

**Fig. 4:**
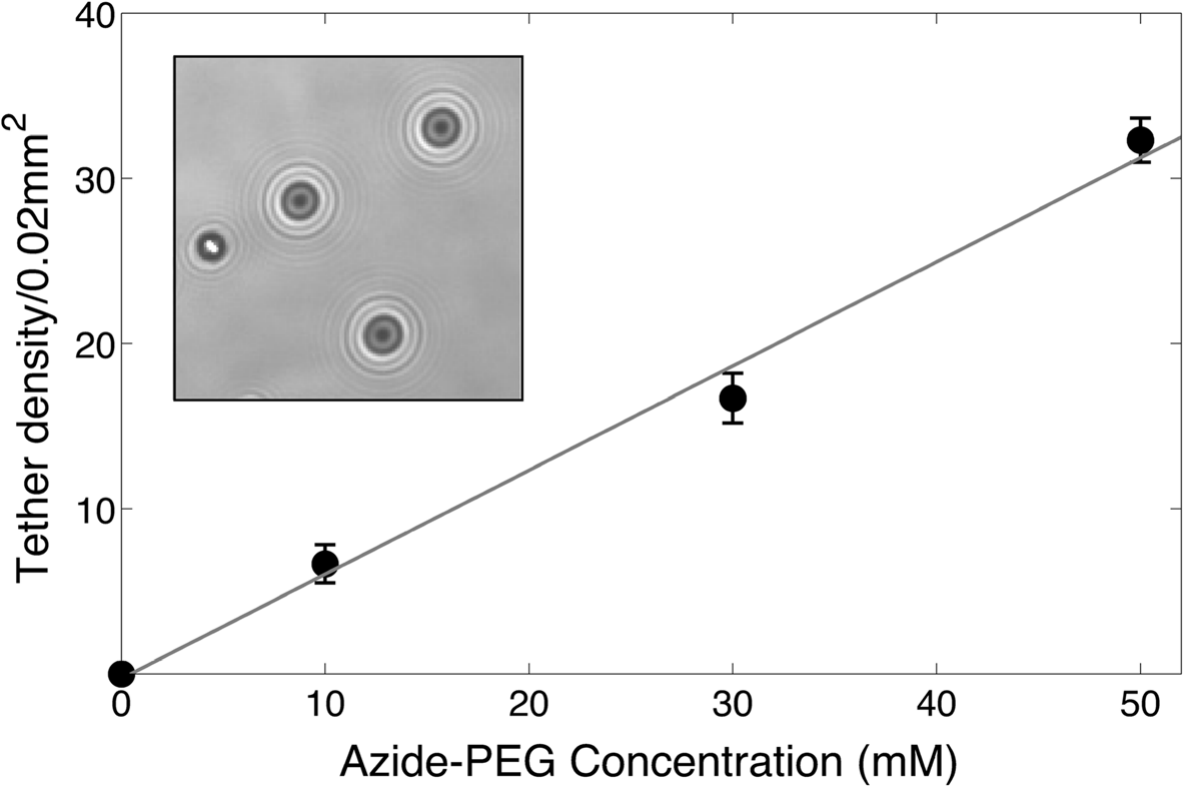
Tether density as a function of PEG concentration. DNA tether density for different Azide-PEG concentrations. The number of tethers increases linearly with increasing PEG concentration. Inset shows an example of a reference bead (left) and three beads that signal 20 kb DNA molecules tethered with click chemistry

The DBCO-functionalized DNA thus covalently attaches to the azide-coated flow cell while the biotin groups at the other end of the DNA attach to the bead. The amount of these DNA tethers is expected to scale with the amount of clickable groups on the surface. To verify the protocol, we varied the density of the azide groups on the surface by using different concentrations of the PEG-linking groups. We determined the tether density by manually counting the number of successful DNA tethers in our field of view (0.02 mm^2^), for different azide-PEG concentrations. As expected, we found that the number of tethers increased linearly with increasing azide-PEG concentrations, see Fig. 4. Importantly, when no azide-functionalized PEG-linkers were added, no tethers of the expected length were observed. This shows that the steps in the protocol are specific and that, conveniently, the tether density is tunable.

**Fig. 5:**
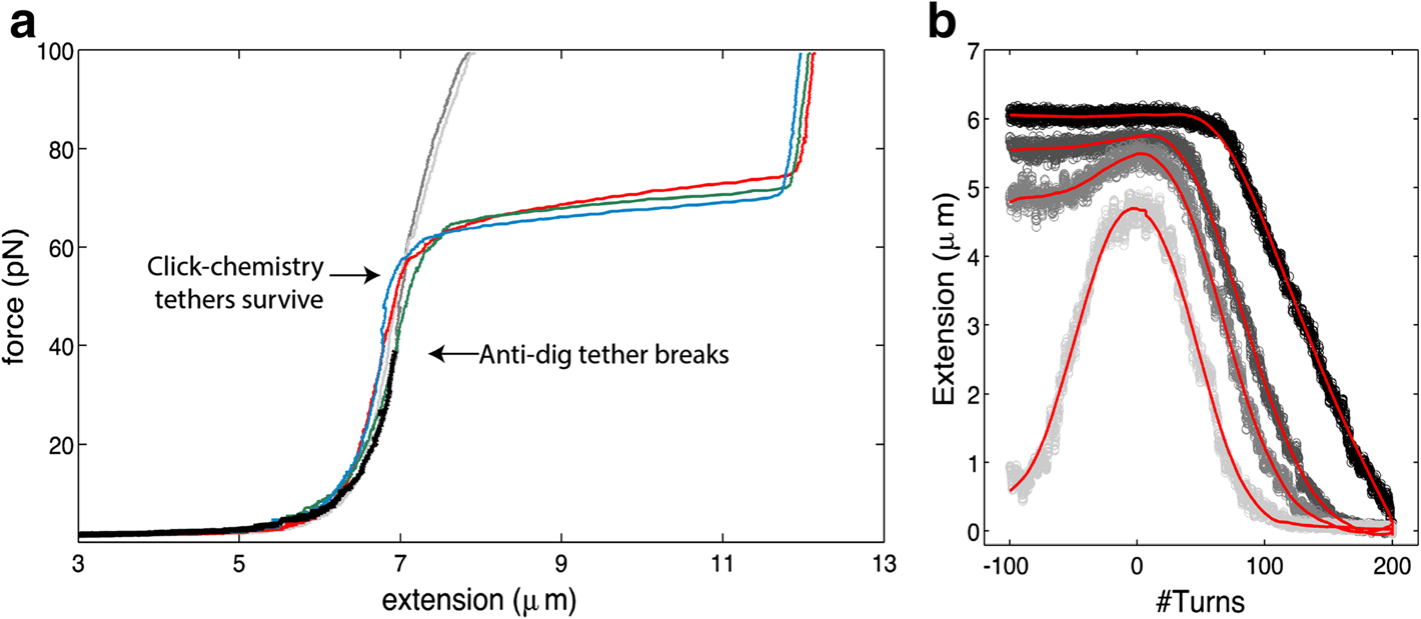
Anchored DNA molecules can be torsionally constrained and withstand forces of >100pN. **a** The DNA molecules anchored with click chemistry show the expected behavior (a strongly rising force, and for unconstrained molecules, a plateau near 65pN as DNA overstretches and a further rise) in a slow force ramp of 1pN/sec. Different colors represent different tethers. All tethers that were bonded by click chemistry withstand forces of over 100pN. By contrast, the DNA anchored with digoxygenin/anti-dig (black) breaks off near 40pN, well before the overstretching point. **b** Rotation curves at constant forces of (light to dark) 0.5, 1, 3 and 5pN, indicating that this 20 kb DNA molecule anchored with click chemistry is torsionally constrained

Our DNA tethers anchored with copper-free click chemistry are able to withstand high force. We anchored 20 kb DNA molecules using copper-free click chemistry and tracked the position of the magnetic beads (corresponding to the end-to-end length of the DNA) while applying a force ramp of 1pN/sec. As shown in Fig. 5, the tethered double-stranded DNA molecules show the expected behavior, viz., with increasing end-to-end distance we observe a strongly rising force, a plateau as the DNA overstretches, and a further rise. As expected, for torsionally unconstrained molecules, overstretching of the double-stranded DNA is observed at about 65pN [34]. Torsionally constrained DNA molecules (depicted in grey in Fig. 5a) are expected to show overstretching at a force of about 110pN, a force that, unfortunately, is just beyond the reach of our set-up [35]. We find an average contour length of 6.75 ± 0.04 μm (as measured from the extension just before the overstretching plateau), indicating correct attachment of the DNA molecules at the functional end groups. Most importantly, the tethers can withstand a force of >100pN (Fig. 5a). The tethers remain stable at this high force for over 12 h, allowing ample time for measurements. By contrast, DNA molecules attached with the conventional anti-dig tag break off well before the overstretching force (cf. the black line in Fig. 5a). In addition, as shown in Fig. 5b, the click-chemistry-assembled DNA tethers can be torsionally constrained, which allows for DNA supercoiling studies with magnetic tweezers. For the described conditions, we found half of the tethers to be coilable. Loss of torsional constrain is likely induced by nicking of the DNA. The new attachment strategy is thus found to be suitable for both high force and torque measurements.

In contrast to the binding of DBCO to azide, the bond between biotin and streptavidin on the other end of the DNA is not covalent. Yet, as can be observed from Fig. 5, this bond also withstands forces of >100pN, which is consistent with earlier reports [17, 18]. For a wide range of applications, the current method, with tethers that contain a mutually orthogonal DBCO/azide bond on one end and biotin/streptavidin on the other, will suffice. Double copper-free click chemistry (with orthogonal click reactions at both bead and surface) can be considered in future applications if even much higher forces are desired.

The copper-free click chemistry attachment strategy presents many advantages. The reaction between DBCO and azide is relatively fast, specific, it does not require a catalyst, and, importantly for some applications, it can be performed in physiological conditions. Furthermore, azide and DBCO groups are relatively small and inert to biological moieties [27] and thus easy to incorporate. There are already numerous examples of the application of SPAAC reactions in biological systems and even living cells [28, 36]. Examples include use of copper-free click chemistry in non-canonical amino acids [37], imaging in live cells [38], joining of DNA strands [39], and DNA-functionalized nanoparticles [40].

Above, we demonstrated the use of a new DNA-attachment method in magnetic tweezers. We note that it can easily be applied to other single-molecule methods as well. For example, in the same manner, polystyrene beads could be coated with click chemistry functional groups for use in optical tweezers. By immobilizing the PEG linkers on the surface, the same copper-free click chemistry can also be used in atomic force microscopy [41], flow stretching and DNA combing.

Single-molecule force spectroscopy opens up the possibility to apply and measure forces on biomolecules, and study DNA-protein interactions. These *in vitro* experiments with bare DNA and purified protein give great insights into the cell machinery, but purified complexes are taken out of their cellular context. As our new method does not cross-react, it is possible to anchor and measure complexes that are directly extracted from cell lysate. Measuring on this native state of biomolecules can be expected to yield new insight into interactions between biomolecules.

## Conclusions

Traditional methods for anchoring biomolecules have encountered limitations in studying DNA-protein complexes in magnetic tweezers related to low force stability and cross reactivity. Here, we developed a method for covalently anchoring biomolecules with copper-free click chemistry, using the reaction between DBCO and azide. This reaction is bio-orthogonal and no catalyst is needed. Furthermore, it is highly specific and it resists high force (>100pN). The protocol is reproducible, fast and uses commercially available reagents. Perhaps most excitingly, covalently linking molecules with copper-free click chemistry opens up the possibility to measure on a wide variety of DNA-protein complexes and complexes isolated from cell lysate.
